# Budget impact analysis of cabergoline for medical treatment of Cushing’s disease in Brazil

**DOI:** 10.20945/2359-4292-2023-0311

**Published:** 2024-07-30

**Authors:** Lukas Fernando de Oliveira Silva, Julia Simões Corrêa Galendi, Manoel Ricardo Alves Martins, Vania dos Santos Nunes Nogueira

**Affiliations:** 1 Faculdade de Medicina de Botucatu Universidade Estadual Paulista Botucatu SP Brasil Faculdade de Medicina de Botucatu, Universidade Estadual Paulista, Botucatu, SP, Brasil; 2 Institute of Health Economics and Clinical Epidemiology Faculty of Medicine University Hospital of Cologne Cologne Germany Institute of Health Economics and Clinical Epidemiology, Faculty of Medicine and University Hospital of Cologne, Cologne, Germany; 3 Departamento de Medicina Clínica e Núcleo de Pesquisa e Desenvolvimento de Medicamentos Faculdade de Medicina Universidade Federal do Ceará Fortaleza CE Brasil Departamento de Medicina Clínica e Núcleo de Pesquisa e Desenvolvimento de Medicamentos, Faculdade de Medicina, Universidade Federal do Ceará, Fortaleza, CE, Brasil

**Keywords:** Cushing syndrome, ACTH-secreting pituitary adenoma, health care costs, budget impact analysis

## Abstract

**Objective:**

The aim of this study was to estimate the budget impact of adding cabergoline to the Brazilian Unified Health System (SUS) formulary for the treatment of patients with Cushing’s disease (CD) who do not achieve disease control after transsphenoidal surgery.

**Materials and methods:**

We conducted a budget impact analysis (BIA) from the perspective of the Brazilian SUS over a 5-year time horizon. We compared two scenarios: ketoconazole (Scenario 1) versus including cabergoline as a treatment option (Scenario 2). All analyses were conducted using Microsoft Excel. Uncertainty was explored in univariate sensitivity analyses.

**Results:**

The total costs were BRL $25,596,729 for Scenario 1 and BRL $32,469,169 for Scenario 2. The budget impact of adding cabergoline to the formulary for CD treatment within the SUS would be BRL $6,091,036 over 5 years. On univariate analyses, variations in the rates of surgical failure and CD recurrence had the greatest potential to affect the final costs associated with cabergoline.

**Conclusions:**

The estimated budget impact of adding cabergoline to the formulary for CD treatment within the Brazilian SUS would be about BRL $6 million. While cost savings cannot be expected, the budget impact of adding cabergoline would be lower than that of adding other treatment options for CD.

## INTRODUCTION

Cushing’s syndrome is caused by a loss of regulation of the hypothalamic-pituitary-adrenal axis, resulting in an inconsistent circadian rhythm of cortisol secretion (1). The prolonged exposure to hypercortisolism may result in various clinical manifestations. Although Cushing’s syndrome is mostly caused by the chronic use of glucocorticoids, it can also stem from an endogenous production of corticosteroids. Approximately 70% of patients with endogenous Cushing’s syndrome have Cushing’s disease (CD) (2), which results from increased ACTH production by a pituitary adenoma and has an incidence of 2-3 cases/1,000,000 inhabitants/year and a prevalence of 40 cases/1,000,000 inhabitants (1).

Individuals with CD have higher morbidity and mortality than those in the general population (3-6). Hypercortisolism leads to comorbidities, especially hypertension and diabetes mellitus, both of which increase the risk of cardiovascular diseases, including acute myocardial infarction and stroke. Patients with CD are also immunosuppressed and more prone to developing infections (3-6).

The first-line treatment for CD is tumor resection through transsphenoidal surgery. The remission rates after transsphenoidal surgery range from 68% to 98% (2) and depend on the neurosurgeon’s experience, technical aspects related to the surgery, tumor extension, and dura mater invasion (7). The recurrence rate after successful transsphenoidal surgery is, on average, 13.4% for microadenomas and 17.6% for macroadenomas. In 5-10 years of follow-up, only 15%-66% of patients remain in disease remission (7,8).

In patients who experience relapse or do not achieve remission after transsphenoidal surgery, other treatment alternatives are available, including repeat transsphenoidal surgery, radiotherapy, bilateral adrenalectomy, and medical treatment. Repeat transsphenoidal surgery is an option particularly in patients with suspected incomplete tumor resection, although panhypopituitarism develops with a considerable frequency in these patients (9). A second treatment alternative is radiotherapy. However, radiotherapy is associated with considerable adverse effects and its effectiveness in reversing hypercortisolism is gradual. A third treatment option is bilateral adrenalectomy, which is reserved for patients who do not respond to radiotherapy but is followed by a daily requirement of glucocorticoid and mineralocorticoid replacement. Notably, bilateral adrenalectomy is associated with an estimated 21% incidence of Nelson’s syndrome, characterized by a triad of cutaneous hyperpigmentation, elevated ACTH levels, and pituitary tumor expansion (10). Therefore, for most patients who experience relapse or do not achieve disease remission after transsphenoidal surgery, medical therapy remains the best treatment alternative (11).

The medications used for medical treatment of CD include ketoconazole, cabergoline, pasireotide, and mifepristone. In Brazil, only ketoconazole is currently listed in the formulary of the Unified Health System (SUS). The effectiveness of cabergoline for patients with CD is mainly supported by observational studies (12). In two retrospective cohorts, cabergoline induced disease remission in 37% of patients over 3-6 months (13) and in 40% at 12 months (14). In a small prospective cohort study, the addition of ketoconazole to cabergoline normalized urinary free cortisol in 6 of 9 patients (67%) (15).

Even though cabergoline has been introduced as a complementary treatment for hyperprolactinemia and acromegaly, it is not available for patients with CD who are covered by the SUS. Based on these considerations, the aim of this study was to estimate the budget impact of cabergoline compared with ketoconazole in patients with CD without disease control after transsphenoidal surgery over a 5-year horizon from the perspective of the SUS.

## MATERIALS AND METHODS

We developed a population data-based model using Microsoft Excel to estimate the impact of cabergoline on the health care budget in Brazil over a 5-year time frame (2022-2026) from the perspective of the SUS. To accomplish that, we carried out a budget impact analysis (BIA) according to methodological guidelines recommended by the Brazilian Ministry of Health (16,17) and following international principles of good practice (18).

The population comprised patients diagnosed with CD who relapsed or failed to achieve disease remission after transsphenoidal surgery. The BIA evaluated the impact of adding cabergoline to the formulary for CD treatment available within the Brazilian SUS by comparing total costs (*e.g.*, direct costs – including medications, follow-up examinations, and procedures) in two scenarios:

(A) Scenario 1: This reference scenario included ketoconazole, the sole medical treatment currently available within the SUS for patients with CD at an average dosage of 800 mg/d (600-1,200 mg/d).

(B) Scenario 2: This alternative (proposed) scenario considered the addition of cabergoline to the formulary for the treatment of CD within the SUS. In this scenario, we assumed that the market uptake of cabergoline monotherapy (*i.e.*, as a replacement for ketoconazole) would increase yearly up to 35% in the 6th year (*i.e.*, corresponding to the proportion of patients expected to respond to monotherapy with cabergoline) while a proportion of patients (*i.e*., 41% in the 6th year) would remain on ketoconazole, based on the proportion of patients expected to respond to ketoconazole. The remaining patients would be offered a combination of cabergoline and ketoconazole.

The proportions representing the market share of cabergoline and ketoconazole were based on results from our study, which employed proportional meta-analysis to assess the effectiveness of medical therapy for CD (19). Table 1 compares the annual market share (*i.e*., the proportion of eligible patients using each medication per year) in Scenario 2. The average dosage of ketoconazole was 800 mg/d (range 600-1,200 mg/d) and the average dosage of cabergoline was 3 mg/week (range 2-4 mg/week).

**Table 1:** Market share comparison for ketoconazole and cabergoline used each as monotherapy and as combined therapy at each study year

Period (year)	Ketoconazole	Cabergoline	Ketoconazole plus cabergoline
2022	87%	10%	3%
2023	74%	20%	6%
2024	66%	25%	9%
2025	58%	30%	12%
2026	41%	35%	24%

### Populational data

**Figure 1:**
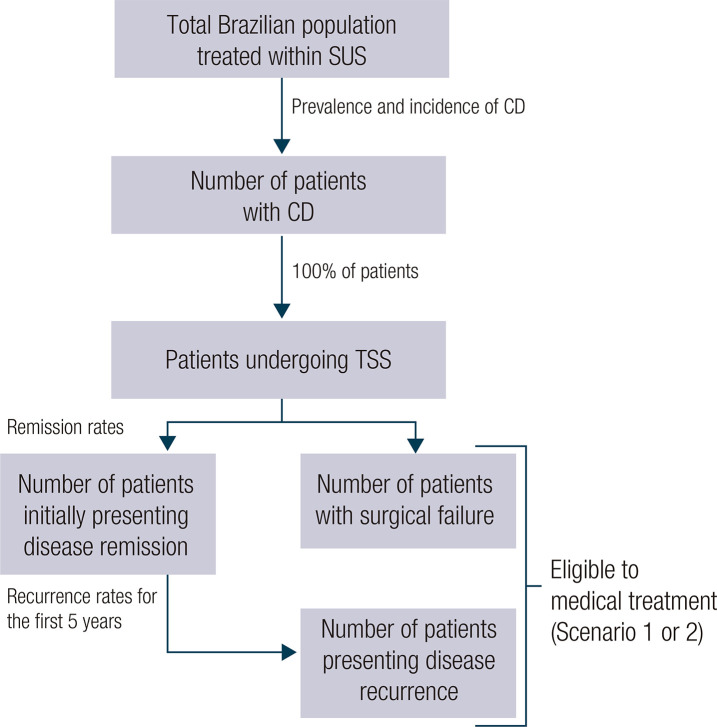
Estimation of the number of eligible patients.

Abbreviations: CD, Cushing's disease; SUS, Brazilian Unified Health Care System; TSS, transsphenoidal surgery.

To estimate the size of the eligible patient population in Brazil, we calculated the yearly number of patients with CD who had recurrence or failure after surgery and subtracted the number of deaths in the period (Figure 1).

We estimated the Brazilian population based on projections from the Brazilian Institute of Geography and Statistics over a 5-year time horizon (Supplementary Table 1). We then calculated the number of patients with CD, considering as a proxy international prevalence and incidence data related to CD retrieved from a systematic review and meta-analysis (20). We assumed that 80% of patients in the estimated Brazilian population would be treated outside the SUS. Since this assumption could not be supported by claims data, we tested a range of proportions in univariate sensitivity analyses.

Next, we used the standard mortality ratio reported by Ragnarsson (21) to estimate the mortality rates for patients with CD (Table 2). Of note, the mortality rate for the general population in Brazil in 2019 was 6.8 deaths/1,000 persons.

**Table 2:** Standard mortality ratio* and mortality rate among patients with Cushing’s disease

	SMR (95% CI)	Mortality rate (95% CI)^†^
Patients with CD		
Remission^‡^	1.9 (1.5-2.3)	12.9 (10.2-15.6)
Recurrence^§^	6.9 (4.2-10.4)	46.9 (28.5-70.7)
General population	2.5 (2.1-2.9)	17 (14.3-19.7)

^*^Estimated by Ragnarsson (21). ^†^Per thousand individuals per year. ^‡^After transsphenoidal surgery. ^§^After surgical failure. Abbreviations: CD, Cushing’s disease; CI, confidence interval; SMR, standard mortality ratio.

Because the standard first-line treatment for patients with CD is transsphenoidal surgery (2), we assumed all patients diagnosed with CD would undergo surgery. Next, to identify the population eligible for medical treatment, we estimated the frequency of recurrence and the number of patients projected to experience surgical failure, both in the prevalent (*i.e.*, patients who underwent surgery before 2022) and incident populations. According to our literature review, the immediate remission rate after transsphenoidal surgery for CD ranges between 71% and 97% (7,22,23). Thus, the rates of complementary surgery after surgical failure range from 3% to 29%.

The average recurrence rate after transsphenoidal surgery for CD ranges from 10% to 26.6% (7,22,23). For patients who underwent surgery before 2022 (*i.e.*, prevalent population), 10%-26.6% were assumed to have remission when entered into the model (Supplementary Table 2). Patil and cols. have shown that the recurrence rates after transsphenoidal surgery increase yearly, but the recurrences usually occur within the first 5 years after surgery (24). Thus, we considered that for patients who underwent surgery in 2022 or later, the proportion presenting recurrence increased over the first 5 years. Table 3 shows the recurrence rates for each study year, as entered into the model. The model assumed similar recurrence rates for microadenomas and macroadenomas (22).

**Table 3:** Recurrence rates over 5 years

Period (years)	Data by Patil and cols. (%)	Relative recurrence^†^ (%)		Recurrence interval (%)
1	0.5	1.9	0.19-0.5	
2	6.7	26.2	2.6-7	
3	10.8	42.3	4.2-11.2	
4	18.1^*^	71	7.1-18.8	
5	25.5	100	10-26.6	

^*^The data for the fourth year was calculated as the midpoint between the third and fifth years. ^†^Calculated based on the relative frequencies reported by Patil and cols. (24).

### Cost data

All direct medical costs (including medications, medical consultations, and laboratory and imaging tests) covered by the SUS were computed without considering the geographic location of the service provider. The resource use in both scenarios is shown in Table 4. In Scenario 2, which included cabergoline, the follow-up costs remained the same except for the frequency of transthoracic echocardiography, which was increased to twice every 3 years.

**Table 4:** Resource use and unitary costs during follow-up

Resources	Resource use for Scenarios 1 and 2 (per year)	Unitary cost^a^
Medication costs		
Ketoconazole 200 mg	1,460 tablets	BRL $0.22
Cabergoline 0.5 mg	312 tablets	BRL $5.83
Medical consultation (endocrinology)	2	BRL $24.90
Laboratory tests (follow-up)		
Blood glucose	2	BRL $7.76
Glycated hemoglobin	2	BRL $2.22
Total cholesterol	2	BRL $7.76
HDL cholesterol	2	BRL $14.72
Triglycerides	2	BRL $14.72
Alanine transaminase	2	BRL $8.43
Aspartate aminotransferase	2	BRL $8.43
Creatine phosphokinase	2	BRL $14.48
Urinary free cortisol	2	BRL $6.10
Complete blood count	1	BRL $17.24
Serum creatinine	1	BRL $7.76
Blood urea nitrogen	1	BRL $7.76
TSH and free T4 (control and/ or late diagnosis)	1	BRL $28.16
Dexamethasone suppression test	1	BRL $25.62
LH	1	BRL $19.13
FSH	1	BRL $16.83
Testosterone	1	BRL $22.25
PTH	0.5	BRL $91.99
Serum calcium	1	BRL $7.76
Total serum protein	1	BRL $5.87
Serum 25-hydroxyvitamin D	1	BRL $30.15
Pituitary magnetic resonance imaging	1	BRL $1127.24
Bone mineral density (spine and/or femur)	0.5	BRL $124.12
Transthoracic echocardiogram†	0.5	BRL $85.19

^a^Corrected for inflation. ^†^In Scenario 2, the frequency of transthoracic echocardiography was increased to twice every 3 years.

Resource use was valued according to the SUS Procedures, Medications, and OPM Table Management System (SIGTAP) with the values corrected to inflation using the Broad National Consumer Price (IPCA) index. The costs of medications were based on a list of maximum prices for sales to the government (25). All cost data are presented in terms of January 2022 values.

### Analyses

An Excel spreadsheet was prepared to aggregate the input data. The total costs for each scenario were calculated by multiplying the costs per patient and the number of eligible patients, considering the variation in population size over the years and penetration of the technology (cabergoline) in the market. The budget impact was calculated by subtracting the total cost of Scenario 2 from the total costs of Scenario 1.

Sensitivity analyses were conducted to quantify uncertainty stemming from input parameters (univariate analyses) and from the assumptions made in framing the BIA (structural uncertainty). On univariate analyses, we tested the ranges for the following input parameters: medication dosage, CD prevalence and incidence, CD mortality, success of surgical treatment, recurrence rate, and cost variations. In structural sensitivity analyses, we considered conservative and aggressive market share progressions (Supplemental Table 3). In the aggressive market share progression, only 36% of the patients would continue ketoconazole and 43% would switch to cabergoline over 5 years, which corresponded, respectively, to the “best expected effectiveness of cabergoline” and “worst expected effectiveness of ketoconazole”. In the conservative market share progression, 46% of patients in 5 years would continue ketoconazole and 27% would switch to cabergoline, also corresponding to the “best expected effectiveness of ketoconazole” and “worst expected effectiveness of cabergoline”, respectively. These proportions were based on confidence intervals that we found in our proportional meta-analysis study on the effectiveness of CD medical therapy (19). Due to the low evidence on the effectiveness of cabergoline for patients with CD, we also calculated the budget impact considering a skeptical market share progression, in which only 20% of patients would respond to cabergoline (Supplementary Table 3).

## RESULTS

The eligible population (*i.e.*, number of patients with CD who had failed surgery or experienced recurrence) comprised 1,932 patients in 2022, 1,945 patients in 2023, 1,957 patients in 2024, 1,968 patients in 2025, and 1,978 patients in 2026. The average cost in Scenario 1 (*i.e.*, the reference scenario considering ketoconazole as a single treatment alternative for patients with CD) was BRL $5,057,256 in the first year, BRL $5,090,086 in the second year, BRL $5,121,225 in the third year, BRL $5,151,250 in the fourth year and BRL $5,176,909 in the fifth year. Over 5 years, the total cost for Scenario 1 would be BRL $25,596,729. In Scenario 2 (*i.e.*, the alternative scenario) the average cost was BRL $5,554,503 in the first year, BRL $6,091,036 in the second year, BRL $6,447,318 in the third year, BRL $6,608,010 in the fourth year, and BRL $7,570,301 in the fifth year. Thus, the total cost for Scenario 2 over 5 years would be BRL $32,469,169.

**Figure 2:**
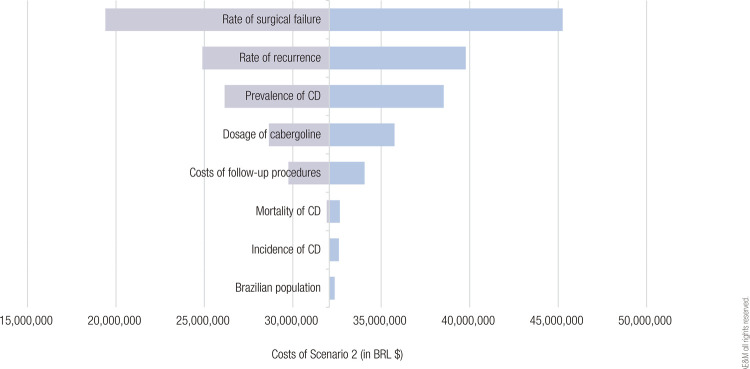
Tornado diagram illustrating the univariate sensitivity analyses of selected variables affecting the total costs in Scenario 2. Abbreviation: CD, Cushing's disease.

The budget impact of adding cabergoline to the formulary for the treatment of CD within the SUS was estimated at BRL $6,872,440 over 5 years. The tornado diagram in Figure 2 shows the results of univariate sensitivity analyses including variables potentially affecting the total costs in Scenario 2. The input variables that most affected the results were the surgical failure rate, CD recurrence rate, CD prevalence, cabergoline dosage, costs of follow-up procedures, CD mortality, and CD incidence.

Considering a more conservative market share progression, the total costs of Scenario 2 would be BRL $32,425,665, and the budget impact of adding cabergoline to the formulary would be BRL $6,828,937. In contrast, in a more aggressive market share progression, the total costs of Scenario 2 would be BRL $33,042,732 and the budget impact would be BRL $7,445,995. Considering a skeptical market share progression, in which cabergoline would be used as monotherapy in only 20% of patients, the total costs of Scenario 2 would be BRL $30,478,410, and the budget impact of adding cabergoline would be even smaller at BRL $4,881,681.

## DISCUSSION

In Brazil, cabergoline is not available for patients with CD who are covered by the SUS. An assessment of the financial consequences of adding cabergoline to the list of medications covered by the SUS was needed. The results of the present study indicate that the budget impact over a 5-year time horizon of including cabergoline in the formulary for the treatment of CD within the SUS would be slightly above BRL $8 million. Because the forecast of the proportion of patients using cabergoline was based on expert opinion, we tested two different market share progressions, one with a more conservative and the other with a more aggressive scenario. We found that the budget impact of adding cabergoline would range between BRL $4.8 million and BRL $11.8 million.

In univariate sensitivity analyses, we identified that the parameters that would most affect the total cost of treatment with cabergoline were the rate of surgical failure and the rate of CD recurrence. In contrast, epidemiological parameters such as the size of the Brazilian population and the incidence and mortality of CD had a smaller impact on costs. Thus, a national registry of patients with CD could be useful to inform future studies on the use of cabergoline and other medical treatments for CD.

The main goal of a BIA is to forecast the financial impact of adding a new intervention and assess whether the new intervention is affordable for the payer. The decision to add cabergoline to the formulary for CD treatment within the SUS depends on the safety and effectiveness of cabergoline, both of which are not assessed in BIAs.

A systematic review and proportional meta-analysis has found that cabergoline monotherapy was associated with disease control in 35% of patients (95% confidence interval [CI] 27%-43%) (19). In contrast, the proportion of patients who achieved disease control with ketoconazole was 41% (95% CI 36%-46%) (19). These pooled proportions of disease control were generated from noncontrolled studies, and thus, a direct comparison of both drugs could be misleading. The only study with a direct comparison of cabergoline *versus* ketoconazole was a small randomized controlled trial of 14 patients (relative risk 0.53, 95% CI 0.15-1.87) (26).

In the present BIA, we only imputed effectiveness data to inform the market share uptake for cabergoline and ketoconazole. Our premise was that patients who do not respond to the clinical treatment with either drug would stop treatment. Notably, the sensitivity analyses considering a lower number of responders in the cabergoline group (such as the conservative and skeptical market share) had a considerably lower budget impact.

Although the certainty of the evidence that cabergoline is superior to ketoconazole is very low, these drugs have very different safety profiles. Cabergoline is mostly associated with nausea and vertigo (19). Adverse events related to ketoconazole include abdominal pain and diarrhea, increase in transaminases, rash, and adrenal insufficiency (27). Thus, patients who do not tolerate ketoconazole due to adverse events remain untreated, and for them, cabergoline may be a promising therapy (28).

Other drugs are available for the treatment of CD, such as metyrapone, pasireotide, and osilodrostat. While the effectiveness of metyrapone for CD is only supported by small case series, the evidence on the effectiveness of pasireotide and osilodrostat stems from large randomized controlled trials. The rate of disease control after pasireotide was 29% (95% CI 25%-35%) in a meta-analysis of two randomized controlled trials including 312 patients (19). However, pasireotide is associated with various adverse events, including diabetes, hyperglycemia, cholecystitis, nausea, abdominal pain, and headache (19). Moreover, one pasireotide dose can cost up to BRL $19,900 (300 µg) and BRL $25,200 (900 µg) and, for the long-acting release formulations, up to BRL $5,600 (20 mg) and BRL $6,500 (60 mg), according to the maximum prices for sales to the Brazilian government (25). Thus, the budget impact of pasireotide would be much higher than that of cabergoline in Brazil. In Finland, the introduction of the long-acting release formulation of pasireotide was estimated to lead to an incremental budget impact of EUR $45,247 in 2018, and up to EUR $231,318 by 2022 for an eligible population of 89 patients with CD (29). In 2012, a BIA conducted in the US estimated that the budget impact of pasireotide would be USD $137,505 in the first year, USD $219,892 in the second year, and U$ 231,954 in the third year after pasireotide launch (30).

Osilodrostat has been evaluated in a randomized placebo study. At 48 weeks, 91 (66.4%, 95% CI 57.9%-74.3%) patients had a complete response, which was maintained for at least 6 months (31). The most common adverse events included nausea (42%), headache (34%), fatigue (28%), and adrenal insufficiency (28%). Notably, osilodrostat has not yet entered the Brazilian market (31).

Some limitations of our BIA must be acknowledged. First, no epidemiological data on CD specific to the Brazilian population were available for the analysis. However, apart from the CD prevalence, other epidemiological parameters had little influence on the total costs of cabergoline treatment and follow-up. Notably, the estimation of the eligible population based on data is more reliable when claims data are available (18). Second, we did not calculate the costs of treating the comorbidities associated with hypercortisolism in CD. However, the effect of cabergoline and ketoconazole on controlling diabetes and hypertension are expected to be similar (11,19) and probably did not affect the results.

In conclusion, cabergoline is an interesting treatment alternative for patients with CD. The expected budget impact of adding cabergoline to the formulary for CD treatment within the Brazilian SUS is estimated at BRL $6 million over 5 years. While cost savings cannot be expected, the budget impact of adding cabergoline would be lower than that of adding other treatment options for CD.

## SUPPLEMENTARY

**Supplementary Table 1:** . Estimated Brazilian population over a 5-year time horizon

Year	Population^*^
2022	214.8
2023	216.2
2024	217.6
2025	219.0
2026	220.3

Source: Brazilian Institute of Geography and Statistics (IBGE). *In millions.

**Supplementary Table 2:** Population eligible for medical treatment in 2021

Population	Prevalent population (number of patients)	Incident population (number of patients)
All patients with Cushing’s disease	8,356-8,378	256-364
Remission	5,933-8,126	182-353
Surgical failure	250-2,429	7-105
Relapse	593-2,161	18-94
Eligible population	843-4,590	25-199

**Supplementary Table 3:** Market share progression per year considering conservative, aggressive, and skeptical scenarios

Period (year)	Ketoconazole	Cabergoline	Ketoconazole plus cabergoline
**Conservative market share progression**			
2022	87%	10%	3%
2023	74%	15%	11%
2024	66%	20%	14%
2025	58%	25%	17%
2026	46%	27%	27%
**Aggressive market share progression**			
2022	87%	10%	3%
2023	74%	20%	6%
2024	66%	25%	9%
2025	48%	35%	17%
2026	36%	43%	21%
**Skeptical market share progression**			
2022	87%	10%	3%
2023	74%	12%	14%
2024	66%	15%	19%
2025	58%	18%	24%
2026	46%	20%	34%
